# The Importance of Modeling Press‐Fit to Accurately Evaluate Interfacial Micromotion as an Indicator of Primary Stability in Uncemented Arthroplasty

**DOI:** 10.1002/jor.70081

**Published:** 2025-10-09

**Authors:** Joshua E. Johnson, Nico Verdonschot, Dennis Janssen, Donald D. Anderson

**Affiliations:** ^1^ Department of Orthopedics and Rehabilitation The University of Iowa Iowa City Iowa USA; ^2^ Orthopaedic Research Laboratory, Radboud University Medical Center, Radboud Institute for Health Sciences Nijmegen the Netherlands; ^3^ Laboratory for Biomechanical Engineering, Faculty of Engineering Technology University of Twente Enschede the Netherlands; ^4^ Department of Biomedical Engineering The University of Iowa Iowa City Iowa USA

**Keywords:** interfacial micromotions, interference press‐fit, plastic bone deformation, primary implant stability, uncemented arthroplasty

## Abstract

Arthroplasty is most often performed to alleviate pain and restore function in patients with end‐stage degenerative joint disease. Uncemented implant fixation is increasingly used in total knee and total ankle arthroplasty. Implantation using interference press‐fit is a manufacturer‐recommended guideline for achieving stable primary fixation in uncemented applications, which is important to prevent long‐term implant failure due to aseptic loosening. However, when evaluating implant–bone interfacial mechanics, many studies have not modeled press‐fit implantation. This can lead to gross underestimation of primary implant fixation stability, limiting the clinical applicability of findings. The goal of this paper is to highlight the importance of simulating press‐fit implantation when evaluating primary orthopedic implant stability using finite element analysis in uncemented arthroplasty. Experiences gained in modeling press‐fit implantation in total knee and total ankle arthroplasty by two different active research groups are shared in this context.

## 1 Introduction

Arthroplasty is most often performed to alleviate pain and restore function in patients with end‐stage degenerative joint disease. Success largely hinges on the attainment and maintenance of stable implant fixation. Total knee arthroplasty (TKA) and total hip arthroplasty (THA) are the most commonly performed arthroplasties in the United States [1]. However, total ankle arthroplasty (TAA) has a greater cumulative annual growth rate than TKA and THA [2]. Surgeons perform arthroplasty using cemented or uncemented fixation, depending upon a variety of patient‐related factors. The use of uncemented fixation in primary TKA increased from 1.9% in 2012 to 20.5% in 2023 [3]. In uncemented applications, primary fixation stability is important to prevent long‐term implant failure due to aseptic loosening, which has been reported to account for 24.4%, 14.6%, and 40% of all TKA, THA, and TAA revisions, respectively [1, 4].

Computational stress analysis methods such as finite element analysis (FEA) are used in concert with complementary benchtop experiments to evaluate orthopedic implant stability. Primary stability performance is typically evaluated by comparing mechanical outcomes such as pull‐off force or implant–bone interfacial micromotions. Implantation using interference press‐fit is a manufacturer‐recommended guideline for achieving stable primary fixation in uncemented applications. However, when evaluating implant–bone interfacial mechanics, many studies using FEA have not modeled press‐fit implantation [5, 6, 7, 8, 9]. Rather, contrary to typical use, line‐to‐line fit has been simulated, with the argument offered that it represents a worst‐case scenario [10]. Not modeling press‐fit implantation can lead to gross underestimation of primary implant fixation stability, which can limit the clinical applicability of findings. For instance, surprisingly high implant–bone interfacial micromotions (as high as 1000 μm, or 1 mm) have been predicted in TAA FEA simulations evaluating the influence of implant design [10, 11].

Stresses/forces generated during press‐fit implantation play a critical role in primary fixation stability. Yet only a few studies have simulated press‐fit implantation when evaluating implant‐bone interfacial mechanics using FEA after TKA [12, 13, 14, 15], THA [16, 17], or TAA [11, 18, 19]. To simulate press‐fit, a geometric interference is virtually created between the bone and implant mimicking the surgical technique. Press‐fit implantation is typically modeled using one of two methods: either by displacing the implant into the bone (matching the physical procedure) [11, 16, 18, 19], or through a contact algorithm‐based adjustment of implant‐adjacent bone elements [12, 13, 14, 15, 18], which generates stresses as the boundary interference is resolved. When press‐fit implantation is modeled in TAA applications using FEA, lower implant–bone interfacial micromotions are observed during physiologic gait loading compared to line‐to‐line fit simulations [11, 18, 19].

The goal of this paper is to highlight the importance of simulating press‐fit implantation when evaluating primary orthopedic implant stability performance using FEA in uncemented arthroplasty applications. Experiences gained in modeling press‐fit implantation in TKA and TAA by two different active research groups are shared in this context.

## 2 Experiences in Total Knee Arthroplasty

Press‐fit implantation has been modeled in TKA using both experimental and computational approaches. The quality of primary fixation and press‐fit implantation of TKA components in experimental and computational analyses are typically evaluated using either pull‐off testing or implant‐bone interfacial micromotions. Pull‐off and pull‐out tests are relatively simple to perform in an experimental set‐up, in which the component is removed from the bone in a displacement‐ or force‐controlled manner. In such a test, the femoral implant is usually moved in the distal direction [15], and the tibial component in the proximal direction [20]. While the pull‐off test does not necessarily represent a physiological loading scenario or a realistic failure mode, it does provide straightforward insight into the quality of the primary fixation. For the femoral component, an adapted loading configuration can also be used, in which the femur is placed in a more flexed position with the compressive load acting on the posterior condyles, representing a roll‐off failure scenario that may occur under high flexion angles [21]. While such a scenario provides a potentially more realistic loading configuration, results are more difficult to interpret due to the interplay of implant features that may promote the posterior condyles being pushed into the femoral bone.

Implant–bone interfacial micromotions are more commonly used to evaluate the primary fixation of TKA components. Micromotions serve as a proxy measure for the capacity of bone ingrowth, with small micromotions potentiating proper ingrowth (facilitating long‐term fixation), and larger micromotions driving potential for the formation of a soft‐tissue layer at the interface, preventing osseointegration. Historically, micromotion thresholds needed for successful ingrowth were identified in animal experiments, with successful osseointegration being achieved when micromotions are smaller than 40–50 µm, and soft‐tissue formation at micromotions exceeding 150 µm [22, 23]. These values have recently been updated to 112 µm for ingrowth and 349 µm for failed osseointegration [24].

Another issue complicating the evaluation of potential osseointegration is the varying definitions of “micromotion” reported in both experimental and computational studies. Experimental measurement techniques include displacement transducers [25, 26] or digital image correlation [27, 28]. The specific measurement technique influences the actual micromotion definition and its values, as it determines the variations in the exact measurement location (e.g., distance from the interface that the measurement is performed), directionality of the micromotions (parallel and/or perpendicular to the interface), and the accuracy or resolution. Moreover, due to limited access, experimental micromotion measurements are typically performed only at the boundaries of the actual implant–bone interface. In computational FEA studies, micromotions are substantially influenced by the adopted definition of micromotion and the type of loading that is simulated. Micromotions can either be reported in the parallel and normal directions separately, or as a total point‐to‐point motion comprising all motion directions. They can be calculated over a single loading‐unloading cycle of a peak load for a specific activity, or over a more complex loading cycle that is composed of multiple loading increments, simulating a more physiological loading configuration [13].

To evaluate primary fixation stability, based on either pull‐off force or implant–bone interfacial micromotions, modeling parameters related to the representation of bone material behavior, interference fit, and the frictional properties at the implant–bone interface are important to consider when simulating press‐fit implantation in TKA applications using FEA (MSC.MARC2007 r1, MSC Software Corporation, Santa Ana, CA).

**Figure 1:**
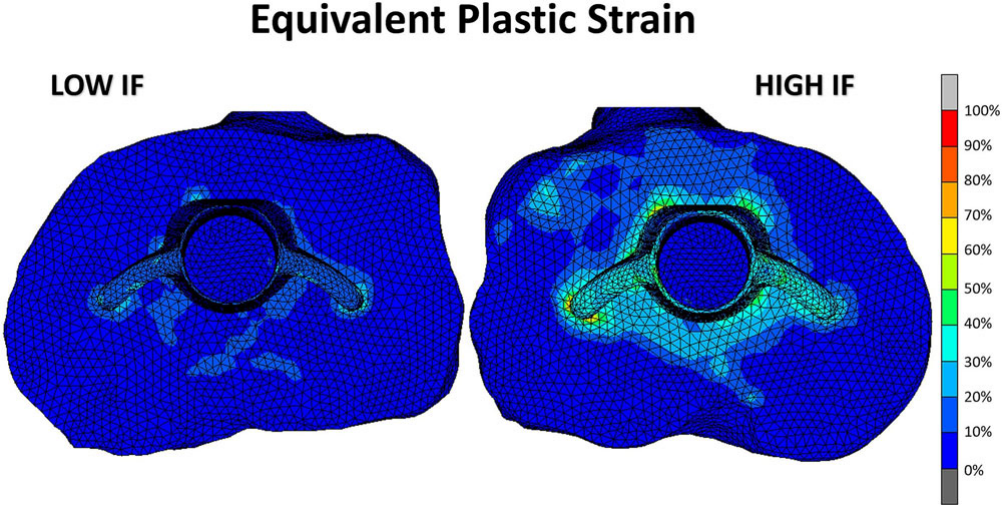
Top view of a tibia after insertion with a tibial TKA component with a low (left, 350 µm) and high (right, 700 µm) interference fit. Increasing the interference fit leads to an increase in plastic bone deformation upon implant insertion. Reproduced with permission from Sánchez et al. [31].

Bone material assumptions are typically based on CT imaging. Heterogeneous material property (e.g., Young's modulus) distributions are assigned based on local CT intensities, which are converted into bone mineral density using a calibration routine [29]. Briefly, a calibration phantom containing tubes with calcium hydroxyapatite of different densities (0, 50, 100, and 200 mg/mL; Image Analysis, Columbia, KY, USA), included in the scan, is used to convert CT Hounsfield Units to *ρ*_QCT_ values. Considering the variability in CT scanning systems and settings, this calibration equation is unique to each scanning session. The *ρ*_QCT_ values are subsequently converted to *ρ*_ash_ values (*ρ*_ash_ = 0.0633 + 0.887 *ρ*_QCT_) [30]. FEA models can either be based on linear elastic bone material property assumptions (*E* = 14,900 *ρ*_ash_^1.86^ MPa) [30], or on material models incorporating nonlinear elastic behavior and permanent bone deformation [30]. While linear elastic bone models can provide information on primary stability, they typically require an adjustment of the interference fit, as a clinically realistic interference fit would lead to excessive stresses in the bone (far exceeding the local yield limit), and subsequently an overprediction of the primary stability. This can lead to a 10–100‐fold reduction of simulated interference fits (e.g., 10–100 µm) [27] compared to those that the implant systems are designed for (1.5 mm/manufacturer guidelines; 1–2 mm range) [12, 15]. This discrepancy can be resolved using plasticity models that allow for permanent bone deformation and damage that occurs during implant placement and impaction. For example, the von Mises plasticity model has been shown to be able to accommodate clinically relevant interference fit magnitudes in femoral [14] and tibial [31] (Figure 1) applications.

While the von Mises material model allows for the simulation of plastic bone deformation, it does not completely capture bone damage that occurs during implant insertion. This phenomenon was investigated in high detail in a prior µCT‐based study. In that particular study, µCT images of the distal femur before and after implantation with a femoral component were compared using digital volume correlation (DVC), which allows for quantification of the permanent bone deformation following press‐fit implantation [32]. The DVC analyses revealed that the plastic deformation of the bone is located in a small region (1–2 mm) near the implant–bone interface, while FEA models using the von Mises model typically show a more extensive pattern of plastic bone deformation.

**Figure 2:**
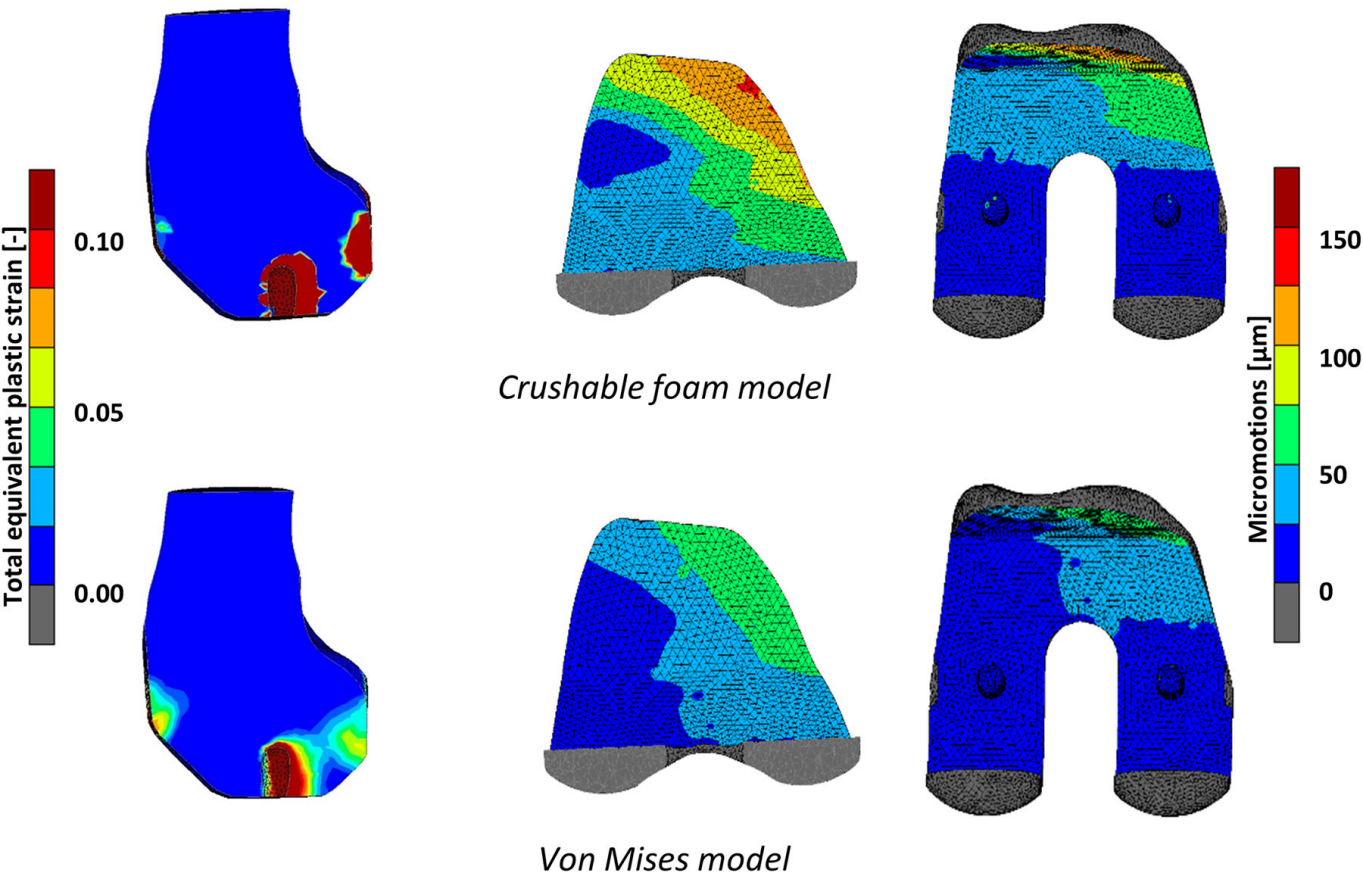
Total equivalent plastic strain (left) and implant–bone micromotions (right) in a model of femoral TKA, simulated using a crushable foam model (top) and a von Mises plasticity model (bottom). Differences are observed between the material models in both the extent of the plastic deformation and the micromotion distribution. Reproduced with permission from Soltanihafshejani et al. [34].

An alternative material model that has been proposed and can potentially reduce the extent of bone plasticity is the crushable foam model. The main difference compared to the von Mises plasticity model is that the crushable foam model incorporates the effect of pressure dependency on the yield surface. Though the complete description is beyond the current scope, most notably, the pressure dependency is represented through a compression yield stress ratio, which can be determined based on a series of hydrostatic and uniaxial material tests [33]. While not extensively validated, the crushable foam material model appears to better capture the mechanism of energy dissipation upon cyclic loading, and has been shown to simulate plasticity closer to the implant–bone interface in femoral reconstructions (Figure 2, left) [34]. This localization of plastic strain more closely resembles the plastic strain distributions that have been observed in DVC analyses of postimplantation µCT scans [32]. Using the crushable foam model also influences simulated micromotions in comparison to the von Mises plasticity model (Figure 2, right), although further investigation is required to confirm whether using the crushable foam model results in micromotion values comparable to experimental findings.

**Figure 3:**
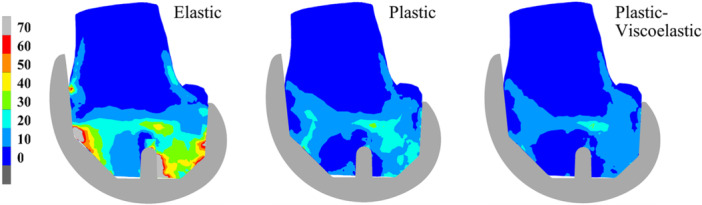
Equivalent von Mises stresses (MPa) at a cross‐section through the lateral condyle after virtual implantation for an elastic, von Mises plastic, and a plastic‐viscoelastic bone material model. Notice the significant decrease in bone stresses when including plasticity, and the additional decrease when including stress relaxation. Reproduced with permission from Gersie et al. [15].

Additionally, though incorporating plastic bone material behavior improves prediction of primary implant stability in press‐fit TKA applications, discrepancies still exist compared to experimental measurements [12], likely due to exclusion of time‐dependent viscoelastic behavior of bone, which may lessen the impact of press‐fit stress/forces over time (Figure 3), potentially influencing micromotions and primary implant stability. In cadaveric femur specimens, pull‐out forces were observed to decrease as early as 30 min after press‐fit implantation [35], and a stress relaxation level of 54% (average) was observed in trabecular bone within 24 h [36]. However, minimal effect on implant stability was observed when combining stress relaxation with bone plasticity in FEA simulating press‐fit implantation of TKA femoral component [15], suggesting a complex interplay between multiple factors (e.g., interfacial surface characteristics and bone abrasion upon implant insertion) contributing to the mechanical response.

As stated above, the representation of interference press‐fit in FEA of TKA depends on the specific bone material model that is chosen. In addition, often an idealized implant‐bone contact interface is assumed, with a line‐to‐line mesh and optimal contact conditions. In clinical practice, however, irregularities are often present at the interface, due to cutting errors, incomplete seating, or a malalignment of the component. Such irregularities also occur in cadaver experiments, which underlines the importance of replicating the experimental conditions as closely as possible when attempting to validate a computational workflow. To some extent, this can be achieved by CT scanning of the bone surface after preparing the bone cuts (providing the initial bone geometry), and by making an optical scan after placement of the implant (providing information on the final implantation). Through registration of these 3D imaging data sets, the obtained reconstructions can be replicated in silico [12].

Another important aspect of realistic modeling of implant–bone interactions in press‐fit TKA is the inclusion of appropriate frictional properties. Typically, literature values are used to select a coefficient of friction that is used in combination with a standard frictional model. These values can vary considerably and may vary with the specific coating material, technology, and surface roughness. Detailed tribological testing of the implant–bone interface revealed that even for two very similar coatings tested against human cadaveric bone specimens, relatively large differences can be seen in the actual mechanical response [37]. Experimental testing also revealed that a substantial amount of elastic deformation occurs even before relative slip occurs at the interface, suggesting that experimental measurements of micromotions may overestimate the actual relative sliding, as they will include elastic deformations in the measurement. This may be important to consider when performing validation studies on primary implant fixation.

Based on applications in TKA, simulating press‐fit implantation taking into consideration bone material behavior assumption, interference magnitudes, and surface geometries and characteristics, is important for evaluating primary implant stability using FEA, to elicit the appropriate physiological response replicating the surgical process. Combined with population‐based modeling approaches that inherently take into account sources of anatomical (age, gender, bone quality, BMI, activity level), implant design feature (implant material, sizing, interference fit, coating properties, level of constraint—posterior stabilized versus cruciate retaining, congruency of articulating surfaces), and surgical parameter (alignment strategy, cutting errors, placement errors) variability [38, 39], realistic simulation of surgical implant fixation may offer broader insights into the effect of specific implant design features and identification of specific risk factors at a population level.

## 3 Experiences in Total Ankle Arthroplasty

Press‐fit implantation in TAA is performed clinically with the goal of achieving sufficient initial stability to enable bony ingrowth. A recent clinical study observed that ~4% of ankles required revision within a 2‐year follow‐up period due to aseptic tibial component loosening after TAA, and that 6.5% of ankles showed tibial lucency around the implant, suggesting that poor osseointegration remains a problem in modern TAA [40]. In addition to implant geometry, interference press‐fit is an important element utilized to achieve stable initial/primary implant fixation in uncemented TAA applications. Physically, press‐fit implantation causes persistent local elastic and plastic deformation of implant‐adjacent bone. The nature of any subsequent bone remodeling at the implant‐bone interface depends upon the local mechanical stimuli (e.g., interfacial micromotions) [22, 41] to facilitate bony ongrowth/ingrowth for long‐term stability.

Many FEA studies of uncemented TAA have not simulated press‐fit implantation when evaluating primary stability [7, 8, 10]. In the absence of press‐fit, computational studies evaluating tibial component stability after TAA have demonstrated peak micromotions in excess of 200 and 1000 μm for implant designs with peg and stemmed fixation features, respectively [10, 42]. Given the importance of press‐fit in facilitating long‐term stability, other recent FEA efforts have focused on more sophisticated modeling of press‐fit and plastic bone deformation to replicate the physical process for the evaluation of TAA initial stability, thereby providing a more meaningful interpretation of implant stability performance in the clinical context.

**Figure 4:**
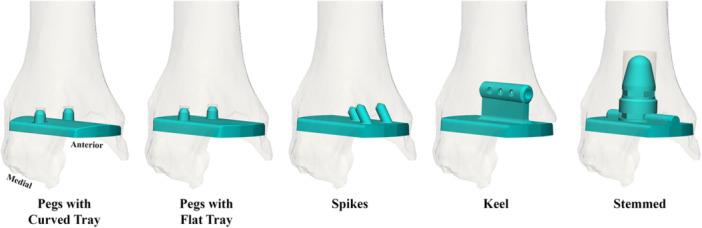
Generalized representations of the TAA tibial component designs modeled, spanning the range of commercially available tibial fixation design features. Lateral tibial sidewall was resected for the keel implant model according to manufacturer guidelines.

The methodology used to evaluate TAA implant–bone interfacial mechanics and implant stability following simulated press‐fit implantation has been described in detail elsewhere [11, 18, 19]. Briefly, the influence of various implant design‐specific and patient‐specific factors on TAA primary stability was evaluated using tibia geometries acquired from anonymized CT scans of four patients with end‐stage ankle arthritis. The same appropriately sized implant tray geometry was used across all patients due to similar distal tibia size. Bone density, inferred from CT intensities, varied between patients at the distal tibia site of implantation. Implant placement followed standard clinical practice, with the location the same across implant designs (Figure 4) to minimize the influence of varying bone density at differing resection heights [10].

Geometric interference was included around implant regions intended for press‐fit fixation according to manufacturer guidelines [11, 18, 19]. Quadratic tetrahedral element meshes were used for all geometries, with element size verified based on a convergence study. A bilinear elastic–plastic bone material model [43, 44] was implemented to allow for any bone yield and plastic deformation during press‐fit and subsequent loading simulations. Isotropic [45], inhomogeneous, patient‐specific material properties were assigned to bone based on CT intensities (0 HU corresponding to 0 g/cm^3^; maximum bone HU corresponding to 1.8 g/cm^3^) [10] and empirical density–elasticity relationships (*E* = 6570 *ρ* ^1.37^ MPa) [46]. Bone yield was simulated using the von Mises yield criterion [30], with an isotropic [12, 30, 44] yield strain of 0.62% [43], and element yield stresses were determined from the yield strain and element moduli. Element postyield modulus was reduced to 5% of its preyield value [43, 44]. First, press‐fit was simulated by displacing the implant into the tibia, with the implant then allowed to elastically recoil after press‐fit, to model the surgical implantation process. Multiaxial forces and moments from the stance phase of gait were then applied to the distal implant surface [47]. Press‐fit displacement and subsequent stance loads were applied through a reference node located at the implant center of rotation, which was kinematically coupled to the distal implant surface [10]. The tibia was held fixed at the proximal end throughout. Implant–bone interfacial contact was modeled using a finite sliding formulation and surface‐to‐surface discretization with default penetration tolerance. Surface normal behavior was modeled using a “hard” contact algorithm with a linear penalty method to enforce the pressure overclosure relationship and contact stiffness automatically calculated. Tangential sliding behavior was modeled using a penalty friction formulation, assuming a titanium plasma spray (TPS) coated implant surface in the base configuration [10].

Simulations were performed in Abaqus Standard (v2018, Simulia, Dassault Systèmes, Vélizy‐Villacoublay, France) using a static analysis accounting for geometric nonlinearity. Automatic time stepping scheme was used with an initial time increment of 0.01 and a maximum time increment of 0.05 for press‐fit simulation. No stabilization schemes were used; however, solution control parameters for time incrementation were set to automatically allow additional iterations prior to convergence check to improve efficiency for severely nonlinear behavior during press‐fit simulation.

To evaluate the influence of press‐fit implantation on initial implant stability, peak micromotions over the entire implant–bone interface were compared between loading simulations of the stance phase of gait after press‐fit or line‐to‐line (i.e., without press‐fit) implantation. Loading simulations with press‐fit implantation were performed with tibial sidewall(s) intact per manufacturer guidelines (Figure 4), while loading simulations without press‐fit implantation were performed with medial and lateral tibial sidewalls resected to represent the least stable fixation scenario. The influence of press‐fit on initial implant stability was evaluated for four implant designs: pegs with curved tray, pegs with flat tray, spikes, and keel (Figure 4) [11, 19]. Additionally, the influence of press‐fit implantation in varying density bone on initial implant stability was evaluated by comparing peak interfacial micromotions throughout stance for a lower‐profile (pegs) and a larger‐profile (modular stemmed) implant design (Figure 4), thereby spanning the range of commercially available tibial fixation design features [18].

**Figure 5:**
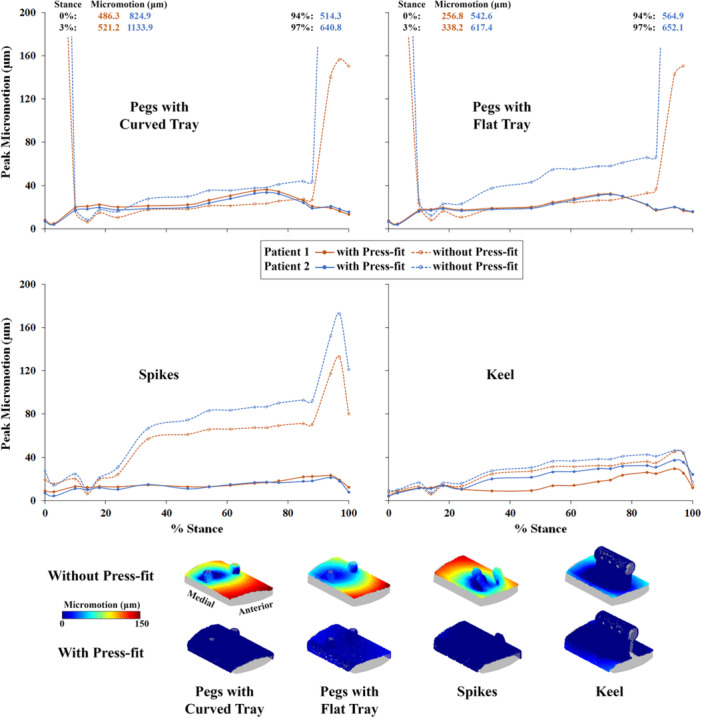
Peak micromotions throughout stance with and without press‐fit simulation for four implant designs and two patients. The *Y*‐axis is truncated at 200 μm for better visualization of curves. The truncated values are provided as text inserts, except for missing instances due to analysis convergence difficulties (at 100% stance for Patient 1 and Patient 2 with both peg implants). Sample micromotion distributions on the implant interface are also shown at 97% stance (bottom), where the majority of maximum peak micromotions occurred.

Implants with larger tibial fixation features were observed to be substantially more stable during gait than lower‐profile implants in the absence of press‐fit implantation with tibial sidewalls resected. In the absence of press‐fit implantation with tibial sidewalls resected, maximum peak micromotions of 521 and 1134 μm (Patients 1 and 2, respectively) were observed during gait for the curved pegs implant design, 338 and 652 μm for the flat pegs design, 133 and 173 μm for the spikes design, and 45 and 46 μm for the keel design (Figure 5). These maximum micromotions occurred either early (3%) or late (94%, 97%) in the stance phase of gait. Intact tibial sidewalls, while providing some constraint in the absence of press‐fit implantation, were only able to contribute to implant stability to a certain extent. In the absence of press‐fit implantation with tibial sidewalls intact, maximum peak micromotions of 212 μm (Patient 1) and 407 μm (Patient 2) were observed for the curved pegs design, and 128 μm (Patient 1) and 368 μm (Patient 2) for the flat pegs design.

When press‐fit implantation was simulated with a 50 μm interference, peak micromotions throughout stance for both patients were observed to be < 40 μm for all implant designs (Figure 5). Maximum peak micromotions when press‐fit was modeled were as low as 36 μm (Patient 1) and 34 μm (Patient 2) for the curved pegs design, and 32 μm (both Patients 1 and 2) for the flat pegs design. For the spikes design, maximum peak micromotions with press‐fit were 23 μm for Patient 1 and 21 μm for Patient 2. Due to the already relatively small micromotions in the absence of press‐fit implantation, maximum peak micromotions with press‐fit (29 μm for Patient 1 and 37 μm for Patient 2) showed the smallest variation for the keel design. Interestingly, peak micromotions varied only by 1.0 ± 0.8 μm (curved pegs), 1.3 ± 1.1 μm (flat pegs), and 0.5 ± 0.5 μm (spikes) on average (±standard deviation) throughout stance when the interference magnitude was increased to 100 μm, and by 1.9 ± 1.8 μm (curved pegs), and 1.7 ± 1.8 μm (flat pegs) when the interference magnitude was increased to 200 μm, compared to the 50 μm interference magnitude.

Several additional simulations were run to explore how the friction associated with a given surface treatment influenced implant‐bone micromotions with press‐fit implantation. The peak micromotions varied by 1.7 ± 1.4 μm (curved pegs), and 1.1 ± 0.9 μm (flat pegs) throughout stance between interfacial contact modeled assuming a TPS (friction coefficient = 0.6) and a 3D printed (friction coefficient = 1.1) surface with 100 μm interference magnitude, and by 1.6 ± 1.4 μm (curved pegs), and 1.3 ± 0.7 μm (flat pegs) with 200 μm interference magnitude.

**Figure 6:**
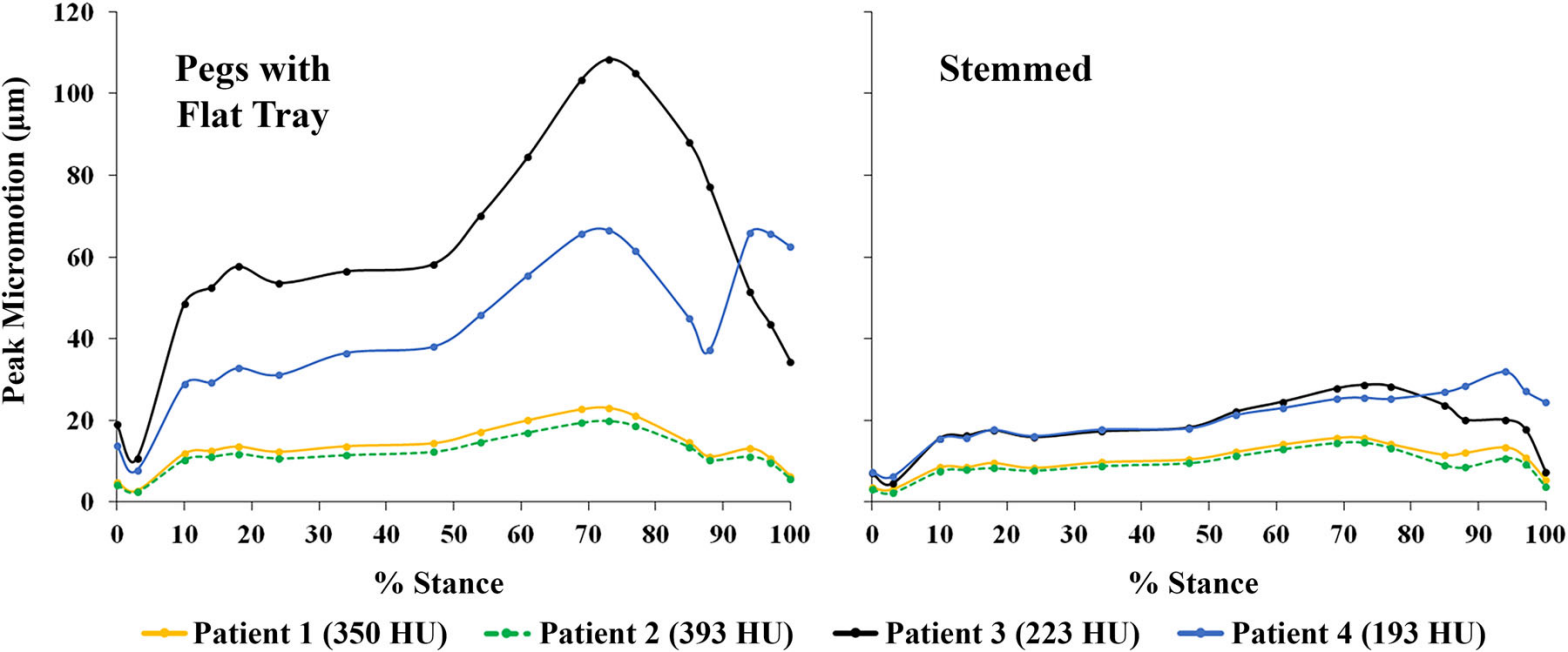
Peak micromotions throughout stance with press‐fit simulation from four patients with varying bone density. Press‐fit was simulated with a 100 μm interference magnitude. Average Hounsfield unit (HU) values are provided for each patient, measured at a region of the distal tibia, 15 mm from the resection height. Adapted from Johnson et al. [18].

Additional simulations were performed to explore the interaction between press‐fit implantation with pegs versus stemmed design and patient‐specific bone density. In the presence of press‐fit implantation, the stemmed implant with the larger tibial fixation features was observed to be more stable during stance in comparison to the lower‐profile implant design, even when implanted in varying density bone (Figure 6). Peak micromotions tended to be larger throughout stance when the lower‐profile design was modeled in patients with relatively lower‐density bone, even when press‐fit implantation was simulated. When press‐fit was simulated, implant–bone micromotions are diminished in correspondence with surrounding bone density [11, 18, 19].

Stresses/forces generated during press‐fit implantation play a critical role in keeping implants stable during subsequent loading. Associated plastic bone deformation, particularly in lower‐density bone, may lessen the impact of elastic recoil accompanying press‐fit, such that other design factors like implant geometry provide complementary stability. Stated differently, interference press‐fit implantation appears to play a more prominent role than implant design in bone that is sufficiently dense, while in less dense bone, a larger‐profile tibial component design with press‐fit implantation appears to be necessary to ensure smaller micromotions [11, 18, 19].

This is important because modern TAA implants are most commonly manufactured with low‐profile designs [40], despite the fact that implants with a larger tibial fixation (i.e., stems, keels) are 95% less likely to fail [48]. Quantifying appropriate bone density thresholds to inform selection of a lower‐profile implant to minimize resection and preserve bone, versus a larger‐profile implant to maximize stability based on patient presentation, warrants further investigation. The modeling of interference press‐fit and associated plastic bone deformation is necessary to realistically replicate the surgical process. Future directions include simulating real‐time bone remodeling taking the influence of biological factors and appropriate mechanical stimulus (e.g., interfacial micromotions) into consideration, to predict success or failure of long‐term osseointegration.

Simulating press‐fit to evaluate TAA primary stability using FEA is not without its challenges. Simplifying TAA implantation mechanics to a line‐to‐line approximation remains an attractive compromise due to additional convergence difficulties resulting from the large bone deformations associated with press‐fit simulation. A potential solution is to model the smallest interference magnitude that provides a reliable estimate of primary implant stability, as the data show implant‐bone interfacial micromotions to be minimally sensitive to interference magnitude (50–200 μm), at least for the comparatively smaller TAA tibial components. Varying interference magnitudes from 250 to 1000 μm have also shown small variations in femoral component micromotions (~10 μm decrease) in TKA [14]. However, interference magnitude may play a role when simulating press‐fit implantation in osteoporotic bone, an area of inquiry requiring further investigation. Moreover, though the data demonstrate that the stresses/forces associated with press‐fit implantation keep the tibial component stable during subsequent stance loading simulations, this is an idealized representation of implant stability performance. In reality, the ankle is typically unloaded up to 4 weeks postsurgery to promote healing, whereby stress‐relaxation accompanying bone viscoelastic response may lessen the impact of press‐fit [15]. Also, upon load‐bearing, tibial component stability is expected to be influenced further with accumulated plastic deformation following consecutive loading cycles, till equilibrium [49]. The influence of these factors on longer‐term implant stability after TAA remains to be evaluated.

## 4 Conclusion

In uncemented application, manufacturers recommend implantation of arthroplasty components using interference press‐fit to achieve stable primary fixation. However, many studies using FEA do not simulate press‐fit implantation when evaluating implant‐bone interfacial mechanics for primary fixation stability performance in uncemented applications. Based on experiences in TKA and TAA applications, simulating press‐fit implantation is essential to accurately evaluate primary implant stability, particularly for the potential use of implant‐bone interfacial micromotions as a mechanical stimulus informing predictions of long‐term osseointegration success or failure.

## Author Contributions

**Joshua E. Johnson:** research design, analysis, data interpretation, drafting the paper. **Nico Verdonschot:** research design, analysis, data interpretation, drafting the paper. **Dennis Janssen:** research design, analysis, data interpretation, drafting the paper. **Donald D. Anderson:** research design, analysis, data interpretation, drafting the paper. All authors have read and approved the final submitted manuscript.
